# Protein tyrosine phosphatases in cell adhesion

**DOI:** 10.1042/BCJ20200511

**Published:** 2021-03-10

**Authors:** Katherine A. Young, Laura Biggins, Hayley J. Sharpe

**Affiliations:** 1 Signalling Programme, Babraham Institute, Babraham Research Campus, Cambridge CB22 3AT, U.K.; 2 Bioinformatics, Babraham Institute, Babraham Research Campus, Cambridge CB22 3AT, U.K.

**Keywords:** cell adhesion, protein tyrosine phosphatases, proteomics, signalling

## Abstract

Adhesive structures between cells and with the surrounding matrix are essential for the development of multicellular organisms. In addition to providing mechanical integrity, they are key signalling centres providing feedback on the extracellular environment to the cell interior, and vice versa. During development, mitosis and repair, cell adhesions must undergo extensive remodelling. Post-translational modifications of proteins within these complexes serve as switches for activity. Tyrosine phosphorylation is an important modification in cell adhesion that is dynamically regulated by the protein tyrosine phosphatases (PTPs) and protein tyrosine kinases. Several PTPs are implicated in the assembly and maintenance of cell adhesions, however, their signalling functions remain poorly defined. The PTPs can act by directly dephosphorylating adhesive complex components or function as scaffolds. In this review, we will focus on human PTPs and discuss their individual roles in major adhesion complexes, as well as Hippo signalling. We have collated PTP interactome and cell adhesome datasets, which reveal extensive connections between PTPs and cell adhesions that are relatively unexplored. Finally, we reflect on the dysregulation of PTPs and cell adhesions in disease.

## Introduction

### Cell adhesion complexes and tyrosine phosphorylation

Cell adhesions are an essential feature of multicellular life and function as both mechanical links between cells and their environment and as major signalling hubs. In addition to maintaining tissue integrity, adhesion complexes must rapidly remodel to accommodate growth, renewal and repair. Reversible protein tyrosine phosphorylation, which expands the static proteome, allows dynamic changes to protein function that can facilitate such remodelling. The coordinated actions of protein tyrosine kinases and phosphatases (PTPs) control phosphotyrosine levels with high spatiotemporal specificity and are both key players in the regulation of cell adhesions. The repertoire of kinases and PTPs has expanded in concert with increased organismal complexity, reflecting a need for greater regulation and specificity [1]. In this review, we will focus on the PTPs and their impact on key adhesive complexes present in human epithelial and endothelial cells.

**Figure 1.**
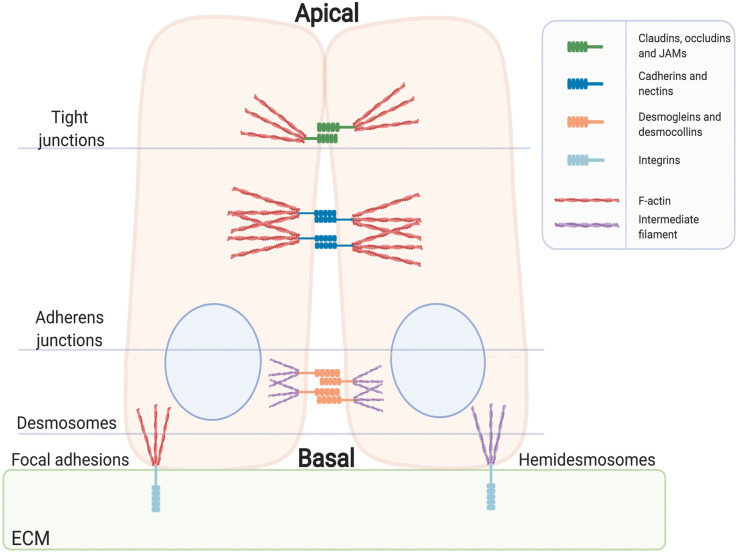
Cell junctions and adhesions in epithelial cells. Tight junctions form apical diffusion barriers. Claudins, JAMs and occludins make up the intercellular plaque and are linked to the ZO adaptor proteins that bind to the actin cytoskeleton. Adherens junctions form basolaterally and are composed of cadherins that form trans homodimers between adjacent cells. The cadherin intracellular domains bind to catenins forming a complex that links to the actin cytoskeleton. Nectins also form part of the adherens junctional structure, and bind to the adaptor proteins Afadin and ZO-1. Desmosomes form structural attachments between cells linked to intermediate filaments. Hemidesmosomes provide a link between the extracellular matrix (ECM), via integrins α6 and β4, and intermediate filaments. Focal adhesions connect the ECM to the actin cytoskeleton. The cytoplasmic region of focal adhesion integrins bind to adaptor proteins such as talin and paxillin, which are linked to the actin cytoskeleton via vinculin. Signalling complexes are recruited to adhesion sites and include adaptor proteins as well as focal adhesion kinase (FAK).

At their core, adhesions consist of transmembrane receptors that couple the cell exterior to the intracellular cytoskeleton, through adaptor proteins. Focal adhesions and hemidesmosomes comprise integrins localised to the basal plasma membrane that bind to extracellular matrix (ECM) components and link to intracellular actin and intermediate filaments (e.g. keratins), respectively (Figure 1). Both complexes function to attach cells to surrounding matrix and must undergo disassembly in order for cells to migrate or differentiate [2,3]. Hemidesmosomal integrins (α6β4)[4] link collagen to intermediate filaments through plectins and BP230 [5]. Focal adhesion integrins are key mechanosensors and can communicate the relative stiffness of the ECM to the cell interior [6]. This is in part mediated by the mechanosensitive adaptors, talin [7] and vinculin [8]. Conversely, they also mediate inside-out signalling in order to balance attachment with motility [9].

Cells are connected to each other through various complexes. Nectins and classical cadherins form heterotypic and homotypic cell junctions connected to the actin cytoskeleton. The classical cadherins bind the armadillo domain-containing β-catenin protein through their short intracellular domains, forming a basic adherens junction complex. α-catenin binding links this complex to the actin cytoskeleton and the δ-catenins, including catenin δ-1 (p120^Cat^), regulate the surface levels of the cadherins [10]. Vinculin can bind to catenins at cell–cell junctions and transmit force to the cytoskeleton, stimulating actin polymerisation [11]. Nectins are connected to actin through the large scaffolding protein Afadin (AF6/MLLT4) [12]. Desmosomes consist of desmoglein and desmocollin cadherin-subfamily proteins and are linked to intermediate filaments, such as keratins, through desmoplakin [13]. They give structural integrity to tissue such as the skin, where they are best studied. The plakophillins are part of the δ-catenin family and have diverse roles, including the regulation of desmosome stability [14]. Finally, tight junctions, comprising occludins, junctional adhesion molecules (JAMs) and claudins, form an apical, selectively permeable barrier between cells and are connected to the actin cytoskeleton through zonula occludens (ZO) family proteins [15] (Figure 1).

All of the complexes described undergo extensive tyrosine phosphorylation of core and peripheral components and are therefore subject to control by kinases and PTPs. We aim to review the growing evidence for the involvement of human PTPs in cell adhesion regulation and how their dysregulation might contribute to disease. We will also discuss their roles in epithelial to mesenchymal transitions (EMT) and associations with Hippo signalling, a key pathway that links adhesion signalling to transcriptional responses. Finally, we highlight how understanding the role of PTPs in cell adhesion may reveal avenues for therapeutic manipulation in cancer and disease.

### Overview of the human PTP family

**Figure 2.**
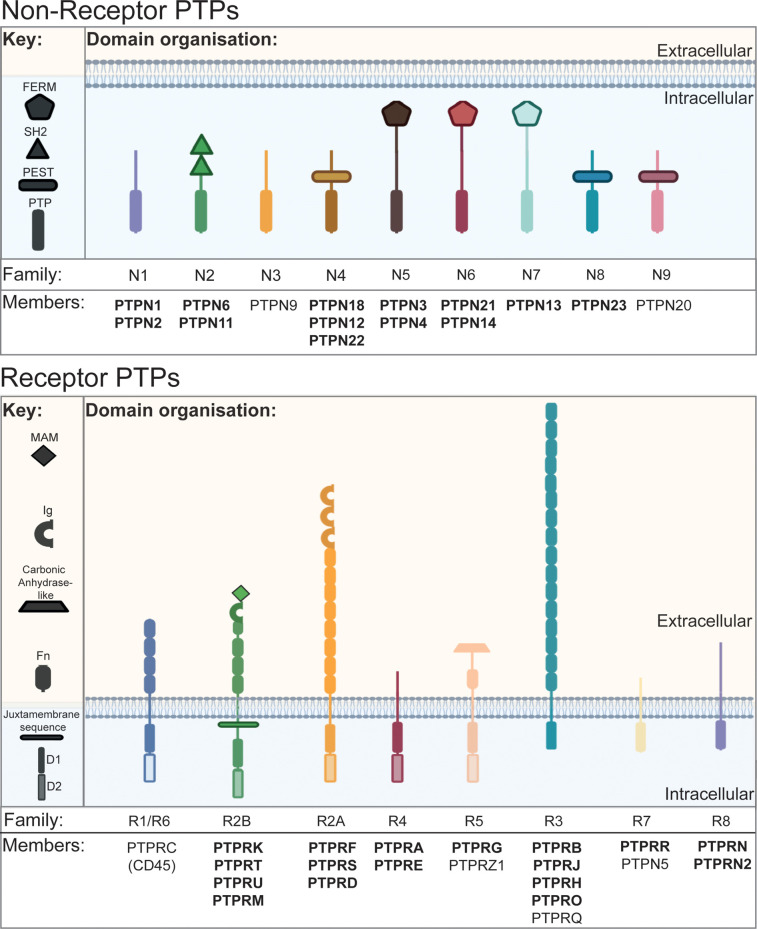
Schematic of the classical PTPs. There are 37 human classical PTPs, which can be divided into 16 non-receptor PTPs within nine subtypes (N1–N9) and the 21 receptor PTPs comprise of eight subtypes (R1–R8). Five out of the eight receptor PTPs have tandem intracellular phosphatase domains; an active membrane proximal D1 domain and an inactive pseudophosphatase D2 domain. The non-receptor PTPs possess additional domains shown in the key. The PTPs in bold represent those that are found in epithelial, endothelial and neuronal cell types and will be discussed in this review.

Phosphatases are a diverse set of enzymes having converged on multiple mechanisms to catalyse the hydrolysis of phosphate bonds [1]. The human classical PTPs are part of a larger family of cysteine-based phosphatases that share a core CX_5_R catalytic motif [16] and includes lipid phosphatases, such as PTEN, and the dual-specificity mitogen-activated protein kinase (MAPK) phosphatases. Beyond this motif, the 37 classical tyrosine-specific phosphatases are defined by additional catalytic motifs [17], including a phosphotyrosine recognition loop that forms a deep pocket that excludes smaller phosphorylated amino acids such as serine and threonine. Besides a phosphatase domain, most PTPs possess additional domains that are critical for their localisation and function [17] (Figure 2). Six of the 37 classical PTPs are classified as pseudophosphatases (PTPRU, PTPRN, PTPRN2, PTPN23, PTPN14 and PTPN21) due to sequence variants in the highly conserved catalytic motifs that predict an impairment or loss of catalytic activity [18]. In addition, 12 of the receptor-linked PTPs (RPTPs) contain tandem phosphatase domains in which the membrane-proximal domain is catalytically active and the distal domain is a pseudophosphatase domain.

PTPs are challenging to study. There is a lack of chemical tools and a requirement for an upstream stimulus for phosphorylation. An additional bottleneck for the field is the identification of direct substrates. Unlike many kinases, tyrosine phosphatases show little specificity at the peptide level, precluding bioinformatic prediction of substrates. Although substrate-trapping mutants have been identified, it is apparent that these do not work consistently for all PTPs therefore empirical tests are required to identify a suitable mutant [19,20]. Even then, substrate-trapping alone does not prove that a protein is a direct substrate. Likewise, modulation of protein phosphorylation by deletion of overexpression of a PTP is also not sufficient to assign substrate status due to potentially indirect or global responses. Once a candidate substrate has been identified it is common practice to purify or isolate a PTP and carry out a dephosphorylation assay. This might be done in-gel [21], in-lysate [22] or *in vitro* [23]. A specificity control should be included within the same experiment, for example, a protein or phosphosite that is not dephosphorylated. Thus, identification of direct substrates should satisfy three key criteria: protein–protein interaction, modulation of tyrosine phosphorylation status in living cells and evidence of direct dephosphorylation. In this review, if we refer to a PTP substrate, we have confirmed that published data meet these three criteria. Where this is not the case we will simply describe the reported effects on particular proteins.

Several PTP families possess domains typically associated with adhesion complexes. For example, RPTPs have extracellular fibronectin, immunoglobulin and MAM (meprin, A-5 protein, and receptor protein-tyrosine phosphatase mu) domains, which mediate adhesion in other cell surface receptors. Furthermore, FERM (4.1 protein, ezrin, radixin, moesin) domains are present in 3 non-receptor PTP families, which typically link transmembrane proteins to the cytoskeleton and are particularly common amongst focal adhesion proteins [24]. Src Homology 2 (SH2) domains, found in the N2 family, are important in building signalling complexes by recruiting and binding to phosphorylated proteins, forming a localised signalling hub [17]. These extracatalytic domains mean PTPs can also regulate signalling pathways as scaffold proteins [25–27]. PTPs have distinct subcellular localisations, as well as cell and tissue-specific expression profiles. For example, the RPTP CD45, a commonly used marker of nucleated haematopoietic cells, has been implicated in cell adhesion processes [28–30]. However, haematopoietic PTPs will not be discussed in this review. For simplicity, we will focus on PTPs expressed in human epithelial and endothelial cell types (shown in bold in Figure 2) and briefly highlight neuronal PTPs involved in axon guidance and synaptogenesis.

## PTPs and cell adhesion complexes

Cell adhesion complexes are formed of a transmembrane receptor and adaptor proteins that couple to the cytoskeleton. These complexes can form between neighbouring cells or anchor the cell to the ECM. Tyrosine phosphorylation can regulate adhesion complexes by inducing conformational changes or facilitating the binding of additional proteins including regulatory enzymes, for example, through phosphotyrosine binding domains. Increased tyrosine phosphorylation can correlate with both cell–cell [31–34] and cell–matrix [35] adhesion formation and disassembly. Adhesion remodelling can be initiated by a number of stimuli such as mechanical force [36], reactive oxygen species (ROS) [37] or growth factors and cytokines [38,39]. There are established roles for focal adhesion kinase (FAK) and Src family kinases (SFKs) in these processes, however, the functions of PTPs are less well defined. Nevertheless, the PTPs function with kinases to tightly control protein phosphorylation and many are important regulators of cell adhesion. Furthermore, the adhesive structures in the extracellular domains of RPTPs mean they are well positioned to sense adhesive cues and couple these to intracellular signalling, an area that remains poorly understood [40].

In evolutionary terms, phosphotyrosine signalling, and particularly PTPs, pre-date multicellularity [41]. Genes encoding phosphotyrosine machinery underwent significant expansion in metazoa [1], consistent with its critical role in the regulation of increasingly complex adhesive processes. The first classical PTPs were orthologs of PTPN1 (PTP1B) and PTPN12 (PTP-PEST) and were present in single-celled amoeba alongside Rho GTPases, integrins and β-catenin, predating classical tyrosine kinases [42]. PTPRF, or LAR, was the first receptor PTP, arising in unicellular choanoflagellates such as *Monosiga Brevicolis* along with the first cadherins [43].

To gain insight into the connectivity between human PTPs and cell adhesion complexes we analysed published proteomics datasets. The use of proximity labelling by BioID [44] and mass spectrometry to define ‘adhesomes' has built up a comprehensive picture of their core components. We collated data from recently reported adhesomes for five major adhesion complexes: tight junctions [45], adherens junctions [46], hemidesmosomes [47], desmosomes [48] and focal adhesions [49,50]. We included an interactome of the mammalian Hippo signalling pathway [51], which links cell adhesion to transcriptional responses. PTP interactomes have been determined using similar proximity labelling as well as affinity-purification approaches coupled to mass spectrometry [22,52,53]. Finally, we included curated PTP-substrate interactions from the human DEPhOsphorylation Database DEPOD [54]. These interactions are detailed in a Supplementary File.

**Figure 3.**
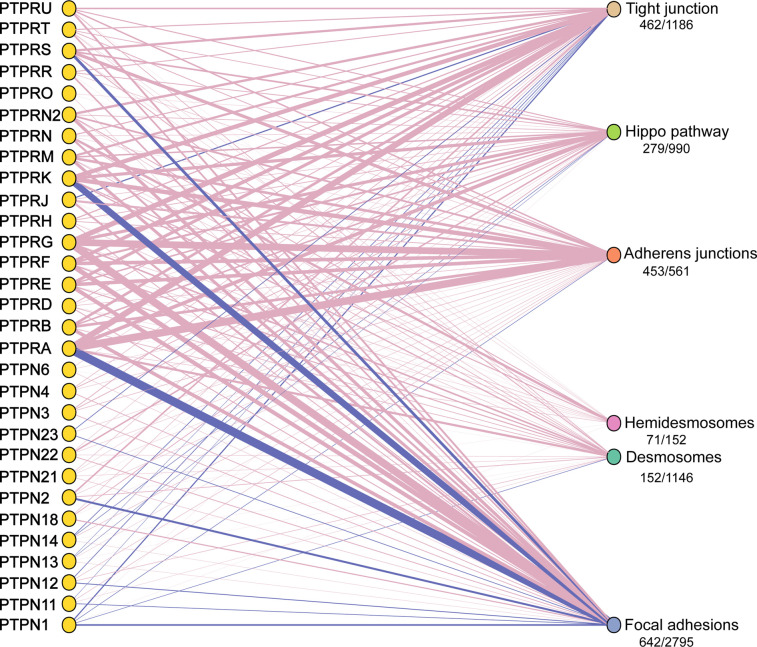
PTP interactions with cellular adhesome components. Published PTP and adhesion complex interactomes were collated. Additionally, we included the interactome of Hippo pathway components and high quality PTP-substrate interactions curated in DEPOD. After removing redundant interactions, a network was generated using the igraph package in R. Pink edges represent a PTP interaction with at least one protein within an adhesome or pathway and blue edges indicate the presence of a PTP within a given adhesome or pathway dataset. The thickness of the edges between nodes represents the number of unique PTP interactions. Ratios beneath adhesome/pathway names indicate the proportion of proteins in each dataset that intersect with PTP interactomes. Full interaction details can be found as a supplement to this review.

Combining these datasets reveals extensive interactions between PTPs and adhesion complex proteins (Figure 3). Several PTPs show connections to multiple adhesion complexes, suggesting they could mediate cross-talk. Furthermore, multiple PTPs are present within adhesomes (depicted by blue lines). This includes PTPN13 (PTP-BAS) and PTPN12 (PTP-PEST) in focal adhesions and PTPRJ in tight junctions. There are established roles for PTPs in cell adhesion (see below), however, this analysis indicates widespread involvement in all major adhesion complexes and Hippo signalling in epithelial and endothelial cells. Although many connections between PTPs and desmosomes and hemidesmosomes exist there are very few functional studies and will therefore not be discussed.

## PTP regulation of cell–cell junctions

### Epithelial adherens junctions

Adherens junctions have the fundamental role of establishing mechanical integrity in animal tissues [55]. They are composed of cadherin–catenin modules that dynamically interact with the cytoskeleton to provide robust adhesions between neighbouring cells [56]. These modules are regulated by tyrosine phosphorylation that can mediate the binding or dissociation of additional proteins. Furthermore, tyrosine phosphorylation can regulate the endocytosis of cadherin–catenin clusters during junction remodelling [57,58], however, the specific mechanism behind this remains unclear [59,60]. Nevertheless, multiple PTPs are required to coordinate the regulation of adherens junctions, in addition to tyrosine kinases such as Eph receptors, epidermal growth factor receptor (EGFR) and SFKs [39,61].

**Figure 4.**
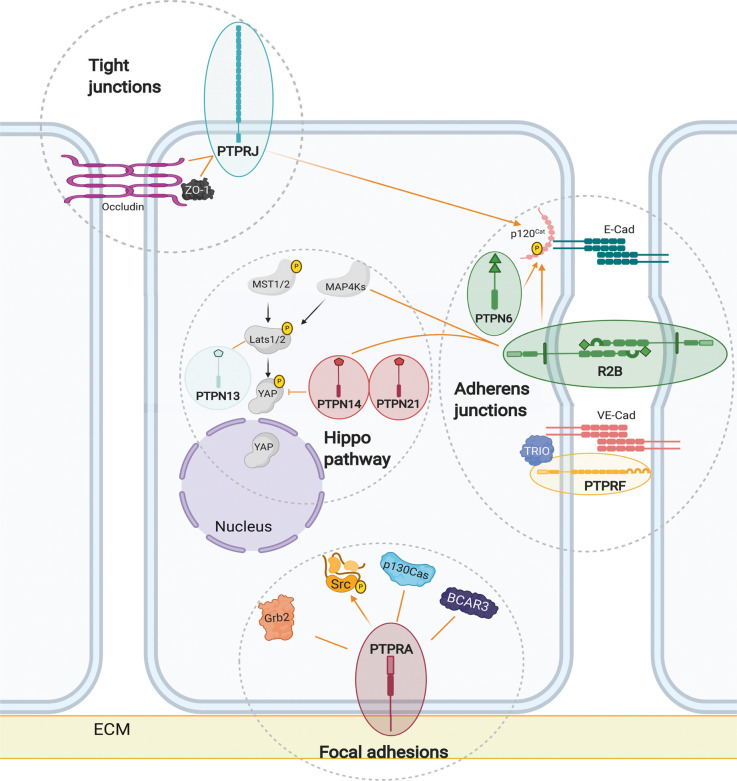
PTP regulation of adhesion complexes and related pathways. Hippo pathway kinases regulate nuclear localisation of the transcriptional co-activators YAP1/TAZ. A direct inhibitory interaction occurs between YAP1 and the non-receptor PTPs PTPN14 and PTPN21. PTPN13 interacts with LATS2, but the functional relevance of this is unknown. A PTPRK substrate-trapping mutant bound the mitogen activated protein kinase MAP4K4/HGK. The receptors PTPRA, PTPRG, PTPRK, PTPRS and PTPRU reportedly interact with PTPN14 and/or PTPN13. Apically, PTPRJ interacts with ZO-1 and occludin at tight junctions. At epithelial adherens junctions, members of the R2B family and PTPN6 interact with p120^cat^; PTPRK and PTPRM can specifically dephosphorylate sites, Y904 and Y228. The R2B receptors can from a homophilic ‘spacer-clamp’ between adjacent cell membranes. At endothelial adherens junctions, NOTCH signalling triggers the formation of a complex between PTPRF, VE-cadherin and TRIO. At focal adhesions, phosphorylated PTPRA recruits a complex of breast cancer anti-estrogen resistance 3 (BCAR3), p130Cas and Grb2 and can dephosphorylate Src at the inhibitory phosphosite Y527.

The cell surface localisation of RPTPs mean they are well placed to regulate cell contacts. For example, PTPRK loss of function leads to decreased junctional integrity in mammary epithelial cells and hyperphosphorylation of human p120^Cat^ at two sites: Y228 and Y904 [22]. PTPRK, and the related receptor in the type 2B family of RPTPs (R2B) PTPRM, can directly dephosphorylate both phosphosites (Figure 4) [22,62]. Phosphorylation of the Y228 site occurs during the formation of adherens junctions [34] and can promote binding between p120^Cat^ and the GTPase, RhoA [63]. Interestingly, increased phosphorylation of this residue correlates with the invasiveness of glioma cells [64]. PTPRJ also reportedly dephosphorylates this site (Figure 4) [65]. Another R2B receptor, PTPRT, which is predominantly expressed in the brain [66,67], has been found to interact with a number of adherens components including E-cadherin, p120^Cat^ and β-catenin [68]. Furthermore, the catalytically inactive R2B paralog, PTPRU, binds to PTPRK substrates including p120^Cat^ and the actin binding protein, Afadin [27] and is proposed to sequester substrates away from related PTPs, including PTPRK and PTPRM, protecting them from dephosphorylation. This suggests that multiple RPTPs can act on the same phosphosite, however, their distinct expression profiles, activities and subcellular localisation will determine their specific roles in cell junction regulation. PTPRA is also required for junctional integrity, but through a distinct mechanism. PTPRA promotes Src activity in epithelial cells. When PTPRA is knocked down, Src substrate phosphorylation is decreased, including cortactin, leading to compromised cell junctions [69]. Finally, our adhesome datasets analysis reveals strong links between PTPRF and PTPRG with adherens junction proteins (Figure 3).

Non-receptor PTPs also regulate cell junctions. For example, SH2-domain containing PTPN6/SHP1 also dephosphorylates p120^Cat^ [70] and following adherens junction disassembly it associates with internalised phosphorylated cadherin–catenin complexes [71]. Its paralog PTPN11/SHP2 can be found associated with vascular endothelial (VE)-cadherin complexes [72] and PTP1B binds to the cytoplasmic domain of cadherins and can dephosphorylate β-catenin [73–75]. Inhibition of PTP1B disrupts E-cadherin adhesion and decreases the transepithelial resistance (TER) of epithelial monolayers [76].

Beyond these experimentally determined interactions between PTPs and individual cadherin and catenin proteins, our analysis of adhesome data indicates that members of all PTP families expressed in endothelial and epithelial cell types interact with at least one adherens junction component (Figure 3). Three RPTPs: PTPRA, PTPRK and PTPRG each had interactions with over 40 adherens junction adhesome proteins. Our analysis of interactome data indicates that the regulation of adherens junctions is finely tuned by an assortment of PTPs, perhaps in response to different stimuli or in different cell types and contexts.

### Endothelial adherens junctions

The endothelium lines the interior of blood vessels. Dynamic cell junctions mediate adhesion between adjacent endothelial cells and comprise VE-cadherin and adaptor proteins such as β-catenin [77]. Blood flow through the vascular endothelium generates fluid shear stress and a small pool of tyrosine phosphorylated VE-cadherin is essential for endothelial mechanotransduction to regulate vessel vasodilation [78]. Endothelial junctions must constantly remodel during angiogenesis, a process which is tightly regulated by tyrosine phosphorylation to ensure correct organisation of new vessels [79–81]. Vascular endothelial growth factor (VEGF) acts as the master regulator of blood vessel function and angiogenesis by binding to VEGF receptors and triggering signalling pathways [82], some of which are mediated by PTPs.

Several PTPs are highly expressed in blood vessels with the receptor PTPRB (VE-PTP) almost exclusively expressed in endothelial cells [66]. Consistent with its expression pattern, deleting or truncating *Ptprb* in mice leads to embryonic lethality due to defects in angiogenesis [83,84]. PTPRB promotes junctional stability in quiescent endothelial monolayers in a phosphatase-independent role by binding and inhibiting the Rho guanine nucleotide exchange factor (GEF), GEF-H1 [25]. The PTPRB-mediated inhibition of GEF-H1 reduced the VE-cadherin internalisation rate by limiting RhoA signalling at adherens junctions. These findings indicate a scaffolding role for PTPRB in vascular adhesion signalling.

PTPRM is also expressed in endothelial cells [85] and localised to the vasculature of all major organ systems [86]. In human pulmonary vascular endothelial cells, PTPRM regulates barrier integrity, possibly through the direct or indirect regulation of VE-cadherin phosphorylation [87]. A *Ptprm* knockout mouse model shows a defect in flow-induced dilation of mesenteric resistance arteries, implicating PTPRM control of the VE-cadherin–catenin complex in the regulation of shear stress mechanotransduction within the endothelium [88]. This could be mediated through its substrate p120^cat^ [22,62]. PTPRF has also been implicated in endothelial barrier function through the formation of a receptor complex with VE-Cadherin and the Rac1 GEF, triple functional domain protein (TRIO) which activates Rac1 and adherens junction assembly [89].

Endoplasmic reticulum (ER)-localised PTPN1 (PTP1B) also regulates endothelial junctions. Deleting *Ptpn1* in mice resulted in enhanced angiogenesis following myocardial infarction [90] and increased skin angiogenesis during wound healing in a type-1 diabetes mouse model [91]. Furthermore, *Ptpn1* expression is up-regulated in a hindlimb ischaemia mouse model which is used to evaluate angiogenesis [92]. Mechanistically, PTP1B catalytic activity may suppress angiogenesis through negative regulation VEGFR2 signalling [92], which normally disrupts VE-cadherin-dependent cell–cell junctions leading to increased proliferation and angiogenesis [93]. In summary, both ubiquitously expressed and endothelium-specific PTPs combine their functions to maintain vascular integrity and support angiogenesis.

### Tight junctions

Tight junctions are vertebrate-specific apical connections between neighbouring epithelial cells that create a selective diffusion barrier to control paracellular permeability [94]. Similar structures are present in endothelial cells, but are not polarised in the same way. The extracellular components of tight junctions are composed of the tetraspan proteins: tricellulin, claudin and occludin, and the single transmembrane proteins, JAMs [15]. These transmembrane proteins interact with a cytosolic plaque consisting of the Par3–Par6 complex and the ZO proteins which link transmembrane components to the actin cytoskeleton [95]. These interactions are key for establishing and maintaining the integrity of cellular apico-basal polarity [96]. Tyrosine phosphorylation of tight junction proteins such as ZO-1, increases tight junction permeability [97]. A small number of tight junction components have well-characterised phosphotyrosine sites and have been reviewed previously [98]. Research on tight junction protein tyrosine phosphorylation has largely focused on the tetraspan protein occludin, which has three major phosphotyrosine sites (Y398, Y402 and Y473) that are phosphorylated by SFKs [99–106]. Mutating these sites alters interactions with ZO proteins and the cSH2-domain of the p85α regulatory subunit of phosphatidylinositol 3-kinase (PI3K), and sensitises cells to ROS-induced barrier disruption [105,106].

PTPN2 (TC-PTP) and PTPRJ have been implicated in occludin regulation. For example, PTPN2 deletion resulted in increased occludin tyrosine phosphorylation in Ang-1-stimulated brain endothelial cells and endothelial hyperpermeability, indicating a role for PTPN2 in blood brain barrier maintenance [107]. Occludin and ZO-1 interact with and are dephosphorylated *in vitro* by PTPRJ, which promotes epithelial cell barrier function through its phosphatase activity (Figure 4) [108]. PTPRJ localises to the apical plasma membrane of epithelial cells [109], and is a component of the tight junction adhesome (Figure 3). However, its large extracellular domain means it is unlikely to be accommodated within the closely apposed intercellular space of the tight junction (Figure 2). This raises questions about how this RPTP regulates tight junctions. One possibility is that PTPRJ dephosphorylates tight junction components to prevent them from becoming ‘active’ in the wrong cellular location or to delimit tight junctions.

Relatively few PTPs have been identified as direct regulators of tight junctions in comparison with adherens junctions and less is known about the role of tyrosine phosphorylation in coordinating tight junction dynamics. Our adhesome analysis suggests a much wider involvement of both receptor and non-receptor PTPs in tight junction regulation (Figure 3).

## PTP regulation of cell–matrix adhesions

### Focal adhesions

Focal adhesions are mechanosensitive, integrin-based adhesions linking the actin cytoskeleton to the ECM allowing the cell to sense and adapt to changes in their immediate extracellular environment [2]. Tyrosine phosphorylation is a key signalling event at the formation and disassembly of focal adhesions [110]. In particular, the tyrosine phosphorylation of FAK recruits a number of different adhesion proteins by providing docking sites for components containing SH2 domains, such as Src [111]. Downstream signalling can alter cytoskeletal dynamics to generate mechanical force for cell motility. It is vital that these pathways are under tight control by tyrosine kinases and phosphatases in order to coordinate the correct responses to internal and external stimuli.

The receptor PTPRF (LAR) reportedly localises to focal adhesions [112]. It also associates with numerous focal adhesion proteins according to proximity labelling experiments, and plays a role in retraction fibre formation during mitosis, a process coordinated by focal adhesion proteins [53]. PTPRF is also a scaffold for the cell-cycle-regulated protein, DEPDC1B, and coordinates focal adhesion disassembly during the transition from interphase into M-phase [113]. Its extracellular domain can interact with ECM components including the laminin–nidogen complex [114] and heparan sulfate proteoglycans (HSPGs) [115]. Furthermore, the Slit- and Trk-like (SliTrk) family protein SliTrk1 functions in neuronal synaptogenesis through an interaction with the PTPRF extracellular immunoglobulin domains [116,117]. A direct link between extracellular binding events and altered phosphatase activity remains to be established. However, PTPRF clustering through the interaction with an intracellular ligand liprin-α interferes with its phosphatase activity [118]. A proposed mechanism by which PTPRF promotes cell-ECM adhesion is through an interaction with c-Abl, triggering Akt signalling and CDK1 activation [119]. Homozygous PTPRF loss-of-function mutations lead to amastia, or absence of breast tissue in humans [120]. Homozygous *Ptprf* knockout mice have revealed this is due to premature involution of the mammary gland [121]. Whether this is due to dysregulation of phosphotyrosine signalling and/or cell–matrix adhesions remains to be determined.

PTPRA is also a key phosphatase needed to coordinate integrin signalling events during the formation of focal adhesions. PTPRA dephosphorylates the inhibitory Src Y527 phosphosite [122,123], relieving autoinhibition, which leads to FAK phosphorylation and the formation of a SFK–FAK complex [124]. Consequently, *Ptpra* null mouse embryonic fibroblasts (MEFs) display impaired cell spreading and migration [124,125]. Interestingly, PTPRA itself is phosphorylated by the SFK-FAK complex on its C-terminal tail at site Y789. This occurs downstream of the initial Src dephosphorylation and is required for cytoskeleton-associated signalling and migration [126]. Phosphorylated PTPRA at focal adhesions recruits a complex of breast cancer anti-estrogen resistance 3 (BCAR3) and p130Cas [127] in addition to Grb2 [128,129] and Src [130] (Figure 4). These data implicate PTPRA as a key regulatory node in focal adhesion signalling, in addition to its role in cell–cell adhesion.

PTPN12 (PTP-PEST) is a focal adhesion regulator recruited to its substrate FAK by PIN1(protein interacting with NIMA 1). It directly dephosphorylates FAK Y397, an autophosphorylation site that facilitates binding to SFKs, phosphoinositide 3-kinase (PI3K) and phosphoinositide phospholipase C gamma (PLCγ). PTPN12 dephosphorylation of FAK reduces the number of focal contacts and consequently increases migration [131,132]. In line with its function at focal adhesions, PTPN12 suppresses glioblastoma invasion [133]. PTPN12 interacts with valosin-containing protein (Vcp) and modulates its phosphorylation at Y805, leading to the segregation of Cas and its subsequent degradation via the ubiquitin- proteasome system. In the absence of PTPN12, Cas is stabilised leading to increased focal adhesion disassembly. Consistent with this proposed function, *PTPN12* mRNA levels are down-regulated in invasive intratumoral regions of glioblastoma [133].

PTP1B has been shown to both promote [134–136] and inhibit [137–139] cell–matrix adhesions depending on the cell type and context. Quantitative phosphoproteomics analysis of *Ptpn1* null MEFs revealed hyperphosphorylated tyrosine residues on a number of adhesion-related proteins, including paxillin and caveolin-1 [140]. Following on from this, studies have validated several of these proteins as PTP1B substrates, including cortactin [141] and EphA3 [142]. PTP1B localises to the ER through its C-terminal tail [143], suggesting it functions at ER-plasma membrane contacts [144]. Indeed, Hernández et al. demonstrated that PTP1B is positioned at paxillin-rich sites of cell–matrix adhesion through extensions of the ER and this localisation aids in maturation and stabilisation of focal adhesions [145]. In addition to the direct dephosphorylation of specific focal adhesion complex proteins, PTP1B can regulate cell–matrix adhesion signalling by dephosphorylating and activating Src, which regulates the Rho GTPase signalling network, influencing adhesion stability and lamellipodial protrusions [146].

In addition to the defined roles for PTPRA, PTPRF, PTPN12 and PTP1B in focal adhesion biology, other PTPs are associated with focal adhesions (Figure 3). In particular, the non-receptor PTPs PTPN2/TC-PTP and PTPN18 have an enrichment of connections with focal adhesions compared with other adhesomes. In fact, six non-receptor PTPs (PTPN2, PTP1B, PTPN11, PTPN12, PTPN23, PTPN9) are found within focal adhesomes (Figure 3) [49,50]. This indicates that a broader role for non-receptor PTPs at focal adhesions is yet to be explored.

### PTP regulation of Rho GTPases and cell migration

Downstream of integrin engagement, the Rho GTPase network is vital for mediating the intracellular responses that regulate cell adhesion and spreading [147]. GEFs promote the GTP-bound, effector-binding state of Rho GTPases that can generate specific signalling responses [148]. In contrast, GTPase-activating proteins (GAPS) promote GTP hydrolysis and signal termination. These GEFs and GAPs are regulated by multiple signalling events including serine/threonine and tyrosine phosphorylation. Several PTPs regulate GEFs and GAPs, either by direct dephosphorylation, as scaffolds or by recruiting other proteins which can modulate their activity [149].

PTPN12 interacts with and modulates the phosphorylation of VAV2, a RhoA and Rac1 GEF as well as p190RhoGAP, a Rho GAP in cells. This is proposed to coordinate VAV2 and p190RhoGAP activities to couple protrusions at the leading edge with tail retraction at the rear, in order to coordinate cell migration [150,151]. p190RhoGAP activity is also linked to PTPN11/SHP2 catalytic activity, indicating a potential regulatory interaction [152]. As highlighted above, PTPRB binds and inhibits the RhoGEF, GEF-H1, leading to reduced RhoA activity at endothelial adherens junction. The exact mechanism of PTPRB-mediated GEF-H1 inhibition is unknown, however, it is proposed to stabilise its inactive conformation, preventing RhoA binding [25]. Finally, PTPRF can bind to the Rac1 GEF, TRIO, but does not dephosphorylate it suggesting a further RPTP scaffold function (Figure 4) [53,153]. In endothelial cells, shear stress can activate NOTCH1 signalling to assemble a mechanosensory complex consisting of PTPRF, VE-cadherin and TRIO. This complex induces Rac signalling and junction assembly to promote barrier function [89]. Thus, PTPs can regulate both the localisation and phosphorylation status of Rho GTPase regulators allowing fine tuning of specific signalling processes that are now being uncovered.

## PTP regulation of Hippo signalling

First described in *Drosophila,* the Hippo signalling has emerged as a key mechanotransduction pathway, linking cell adhesion to transcriptional responses [154]. Fundamentally, the pathway controls the nucleocytoplasmic shuttling of the transcriptional co-activators, YAP1 and TAZ. When the core kinases of the pathway are active, YAP1/TAZ are phosphorylated leading to their retention in the cytoplasm. However, when the kinase portion of the pathway is ‘off’, dephosphorylated YAP1/TAZ accumulate in the nucleus where they can drive expression of genes involved in cell proliferation and survival (Figure 4) [155]. The pathway is regulated by cell density and extensively linked to cell junctions and cytoskeletal dynamics [156]. Given their extensive roles in cell adhesion, the PTPs are ideal candidates to regulate Hippo signalling.

PTPN13 and PTPN14 are present in the Hippo pathway interactome (Figure 3) and PTPN14 is functionally linked to the pathway. YAP1 directly interacts with a PTPN14 PPxY motif, through its WW domain. This interaction sequesters YAP1 in the cytoplasm, inhibiting its transcriptional co-activator function in a cell-density dependent manner [157,158]. The PTPN14 paralog, PTPN21, can also bind to YAP1 and limit its activity [159]. PTPN13 interacts with LATS2, a core Hippo pathway kinase, but the functional consequences are unknown (Figure 4) [51].

Several RPTPs had enriched interactions with the Hippo pathway including PTPRK (Figure 3). PTPRK can form a substrate-trapping interaction with mitogen-activated protein kinase kinase kinase kinase 4 (MAP4K4, or HGK) [22], a component of the expanded Hippo pathway [160], raising the possibility that PTPRK is an upstream regulator (Figure 4). Five RPTPs (PTPRK, PTPRG, PTPRA, PTPRS and PTPRU) were found to interact with PTPN13 and/or PTPN14 suggesting the involvement of a complex PTP network in the Hippo signalling regulation (Figure 4). Indeed, the membrane-bound RPTPs are well positioned to act as signal transducers of mechanical cues generated from changes in cell adhesion.

## Regulation of PTPs at cell adhesions

We have described how PTPs regulate cell adhesions, however, cell adhesions can also feedback to PTPs to control their activity and localisation. This is particularly true for cell surface RPTPs. For example, the R2B receptor family form extracellular homophilic interactions that stabilise the receptors at cell–cell contacts [22,161–164]. Following structural studies on the extracellular domain of PTPRM, it was suggested that the rigid trans dimers could act as a ‘spacer-clamp’ between adjacent membranes, locking receptors into a specific location to allow spatiotemporal regulation of local substrates (Figure 4) [165]. Intriguingly, the predicted extracellular domain length of PTPRM (∼30 nm) is longer than the anticipated intercellular distance for a mature E-cadherin junction (∼18.5 nm) [166]. Thus, it might be expected that R2B receptors are spatially separated from mature adherens junctions, which has been observed by super-resolution microscopy [22]. This suggests that these receptors may function primarily in assembly and disassembly of junctions due to their exclusion from mature junctions.

PTP oxidation is another regulatory mechanism that can occur at cell adhesions. Reactive oxygen species (ROS), such as hydrogen peroxide (H_2_O_2_), are important regulators of wound healing [167–169]. There is also growing evidence that ROS act as mediators of cell migration and cell–cell contact formation [170]. PTPs are well-established targets of cellular oxidation [171]. Their redox sensitivity is conferred by the reactive catalytic cysteine within the conserved HC(X_5_)R motif [172], which is important during nucleophilic attack on phosphate bonds but also renders it sensitive to oxidation and inhibition [173]. PTPs can undergo diverse reversible modifications upon oxidation, including disulfide bonds and adduct formation [174,175]. In a cellular setting, PTP oxidation can occur in response to various stimuli, including the local production of extracellular superoxide by plasma membrane-localised Nicotinamide adenine dinucleotide phosphate (NADPH) oxidase (NOX) enzymes, in response to activation of cell surface receptors by ligands such as epidermal growth factor (EGF) and transforming growth factor (TGF)β [176]. Superoxide dismutase (SOD), converts superoxide to H_2_O_2_, which is thought to enter cells through aquaporins [177]. In addition, focal adhesion formation is accompanied by a significant but transient increase in H_2_O_2_ production [178]. It has been postulated that this influx of H_2_O_2_ may act to inhibit PTP catalytic activity thereby promoting the phosphorylation of downstream integrin mediators, such as FAK [178,179]. ROS production at focal adhesions in endothelial cells involving the NOX subunit p47*^phox^* was proposed to oxidise PTPN12 (PTP-PEST), downstream of Rac GTPase activation [180]. p47*^phox^* was recruited to focal adhesions by scaffolding proteins Hic-5 and TRAF4. Hic-5 had previously been shown to recruit PTPN12 to focal adhesions [181]. PTPN12 inactivation at focal adhesions was speculated to act in a positive feedback loop to promote activation of Rac and initiate membrane ruffling.

Rac is a known NOX activator [182], promoting ROS production in response to various stimuli including cytokine stimulation [183] and cell contact formation [184]. The activation of Rac1 has been shown to promote α-catenin phosphorylation via ROS production resulting in loss of cell–cell adhesion [185]. Interestingly, as the PTPs increasingly become recognised as key regulators of Rho GTPase GEFs and GAPs, the Rac1-mediated production of ROS suggests a potential additional layer for regulation through PTP inactivation. The acute control of PTPs by oxidation adds further complexity to their spatiotemporal regulation and potential functions at cell adhesions, particularly as PTPs show distinct susceptibilities to oxidation [186]. Furthermore, PTP oxidation exhibits cell and tissue-specific patterns, which change with age, at least in mice [187,188]. This raises the possibility that cell adhesions may become dysregulated with age due to altered PTP oxidation.

## PTPs in the nervous system

During the development of the nervous system, cell adhesion is critical for promoting neurite connections and several PTPs have been implicated in regulating this process. PTPRM has been shown to regulate N-cadherin-dependent neurite outgrowth [162] and this is important during retinal development [189]. The type 2A family of RPTPs (R2A): PTPRF, PTPRD and PTPRS function in axon guidance, partly through interactions with diverse postsynaptic proteins. The function of the R2A receptors in synaptic organisation has been reviewed very recently [190], therefore we will only provide a brief summary.

The role of R2A RPTPs in axon guidance was first characterised in invertebrate systems by genetic studies. Leukocyte common antigen–related (dLar) is the *Drosophila* orthologue of the three mammalian R2A receptors and is highly expressed in developing neurons. DLar is critical for motor axon guidance decisions [191] and for the formation of neuromuscular junctions during synaptic development of larvae [115,192]. A number of dLar interactors are also important in *Drosophila* axon guidance*,* including Trio [193], Liprin-α [192] and Caskin [194]. Recent work on neural circuit wiring in *Drosophila* identified immunoglobulin superfamily cell adhesion molecule (CAM) Sticks and Stones (Sns) as a novel ligand for dLar [195]. The authors also demonstrated that PTPRD and PTPRF can bind to the human orthologue of Sns, Nephrin. Interestingly, Nephrin is a major component of the specialised intercellular contacts that form between kidney podocytes known as the slit diaphragm [196]. The interaction between the R2A receptors and Nephrin indicates a potential role in regulating specialised cell junctions. PTP-3 is the sole R2A family orthologue in *C. elegans* and has two isoforms: PTP-3A and PTP-3B, which differ by length of the extracellular domain [197]. The two isoforms have been shown to have distinct roles; PTP-3A localises to presynaptic domains and contributes to synapse development, whereas PTP-3B functions extra-synaptically in axon guidance [198].

In vertebrates, the R2A family enact three general roles; mediating cell–cell adhesion at synapses, regulation of presynaptic differentiation and supporting postsynaptic differentiation [199]. The R2A receptors bind to chondroitin sulfate proteoglycan (CSPGs) and HSPGs, which are known to regulate axon guidance. Recently, the glycan sulphation patterns of HSPGs and CSPGs were shown to determine whether axonal growth is promoted or inhibited. This is proposed to be mediated by binding and modulating PTPRS activity. Heparan sulfate octa-saccharides promote ligand-induced PTPRS clustering that inhibits its phosphatase activity. Conversely, binding to tetra-saccharides such as chondroitin Sulfate promotes catalytically active PTPRS monomers [200]. A role for R2A family members in synaptic organisation in humans is supported by exonic single nucleotide polymorphism associations with several neuropsychiatric disorders such as restless leg syndrome [201] and ADHD [202].

Finally, there are emerging roles for the type 3 RPTPs (R3 family) in the field of axon guidance, in particular PTPRJ and PTPRO. Members of the R3 family are able to interact with and dephosphorylate Eph receptors, which coordinate axonal projections [203]. A recent study found PTPRJ may be a key regulator of retinocollicular projections in mouse retinas. PTPRJ expression is up-regulated during retina development and *Ptprj* knock out mouse retinas have misrouted axons. PTPRJ function in axon guidance is associated with the regulation of repulsive signals from Ephrin and c-Abl activities [204]. Thus, several RPTPs are important coordinators of axon guidance and synaptic adhesion.

## PTPs in cancer

Dysregulation of protein tyrosine phosphorylation is often a key driving event in oncogenesis leading to uncontrolled proliferation and cell survival. Despite functioning as negative regulators of tyrosine phosphorylation, PTPs can act as both tumour suppressors and oncogenes [205]. The likely tumorigenic outcome of dysregulated PTPs that control cell adhesion is increased invasiveness and metastasis, a key hallmark of cancer [206]. Consistent with this, numerous PTPs have been implicated in the regulation of EMT. Aberrant EMT can lead to metastasis, contributing to cell dissemination from the primary tumour and colonisation of a new site. EMT is a reversible process in which epithelial cells lose their apical-basal polarity and loosen cell–cell contacts whilst undergoing an extensive reorganisation of the actin cytoskeleton to form a more spindle-like mesenchymal cell [207]. Cells in a mesenchymal state exhibit increased focal adhesions, and enhanced motility and invasiveness [208]. EMT is increasingly recognised as a continuum with intermediate states and cells displaying considerable plasticity [207]. A hybrid EMT cell state might be the most tumorigenic, having increased invasiveness compared with epithelial cells, while preserving the ability to colonise metastatic sites [209,210].

**Figure 5.**
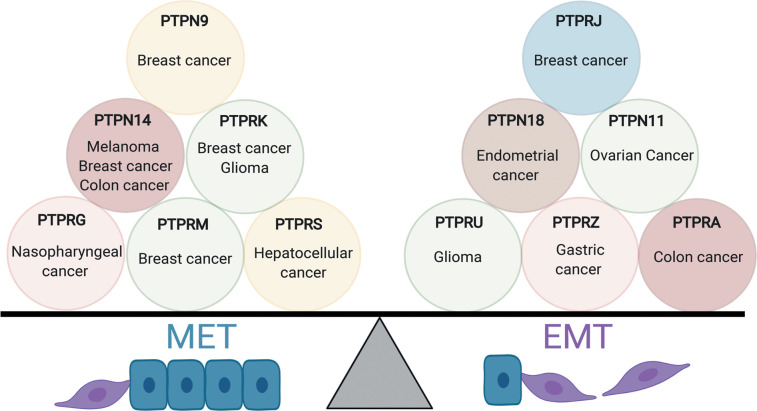
PTPs control epithelial to mesenchymal plasticity in cancer cells. Changing the expression or activity of specific PTPs that control cell adhesions in different tissues or cell lines tips the balance towards a more epithelial or mesenchymal phenotype. In cancer this could manifest in metastasis-inducing behaviours.

There are several examples where RPTP loss of function leads to increased cell motility and invasion including PTPRM and PTPRK in breast cancer [211,212], PTPRG in nasopharyngeal carcinoma [213] and PTPRS in hepatocellular carcinoma [214]. PTPRK was recently highlighted as a regulator of the EMT hybrid state [215], consistent with previous findings where PTPRK KO cells exhibited reduced junctional integrity and cell height, but did not undergo a full transition to a mesenchymal state [22]. PTPN9 overexpression suppressed invasion in breast cancer [216] and PTPN14 overexpression correlates with decreased invasion in several cancer cell lines [217]. In melanoma cells, PTPN14 interacts with and negatively regulates caveolin1 (CAV1) phosphorylation, preventing Rac1-mediated invasion [217]. PTPs can also promote invasion including PTPRU in glioma [218], PTPRJ in breast cancer [219], PTPRA in colon cancer [220] and PTPRZ in gastric cancer [221]. PTPRA regulates cellular contractility through SFKs and myosin light-chain kinase (MLCK) and its presence is required for colon cancer cell invasiveness [220]. PTPN18 is required for invasion in endometrial cancer cell lines [222] and PTPN11/SHP2 enhances the invasion and metastasis of ovarian cancer cells [223]. Thus, distinct PTPs can promote EMT, or the reverse process MET, suggesting a dynamic balance within the cell that can be tipped by altered PTP expression or activity (Figure 5).

PTPs are implicated in cancer through their cross-talk with Hippo signalling, which has been linked to solid tumour initiation and growth [224]. Indeed, YAP1 is an oncoprotein in several cancer types [225]. PTPN14 is a tumour suppressor in pancreatic cancer through inactivation of YAP1, functioning downstream of p53 [226]. Multiple somatic PTPN14 mutations have been associated with breast cancer, colon cancer and skin basal cell carcinoma suggesting an additional tumour suppressive role in preventing over-activation of the Hippo pathway in these cancer types [227–229]. The diverse roles of PTPs in cancers warrants further study in order to identify potential therapeutic targets downstream of tumour suppressive PTPs, or to target those that promote cancer states.

## PTPs as therapeutic targets

PTPs are important therapeutic targets in a number of disease settings [230,231]. It has historically been challenging to identify specific, bioavailable PTP inhibitors, due to their positively charged active sites and substrate recognition through specific protein–protein interactions, rather than at the peptide level. However, allosteric inhibitors have shown promise [232–234]. We will review the PTPs that are targets based on their roles in adhesive processes.

The receptor PTPRS is a target in spinal cord injury. PTPRS binds to extracellular CSPGs, which inhibit sensory neuron outgrowth during regeneration [235]. Deleting PTPRS in sensory neurons improves neural regeneration [235]. In contrast, high concentrations of PTPRS trap growth cones in a dystrophic state [236]. A peptide of the PTPRS intracellular wedge domain that was designed to prevent PTPRS clustering and administered to injured rats led, to promising serotonergic innervation to the spinal cord injury and subsequent recovery of the locomotive and urinary systems [236]. However, this is at odds with the recent suggestion that it is PTPRS catalytic activity that leads to impaired neural regeneration by dephosphorylation of cortactin [200]. The wedge domain of certain PTPs was previously described as a key regulator of PTPRA dimerisation and activity [237]. However, the LAR/PTPRF crystal structure did not indicate a role for the wedge domain in interdomain contacts [238]. Similarly, the orthologous region in PTPRM is also not involved in dimer contacts [239]. Thus, the use of wedge domains to inhibit PTPs remains a controversial area.

Patients with cardiovascular diseases can exhibit compromised VEGF/VEGFR2 signalling resulting in impaired angiogenesis following injury [240]. A cell-permeable allosteric PTP1B inhibitor promotes endothelial cell migration by activating the DOCK180/Rac1 pathway without the requirement of VEGFR2 activation. This inhibitor could potentially be used to accelerate re-endothelialisation after vessel injury in these patients [241]. Also related to control of endothelial cells, the PTPRB-active site targeting inhibitor, AKB-9778, suppresses ocular neovascularisation and excessive vascular permeability in mice by activating the endothelial receptor tyrosine kinase Tie2, a reported target of PTPRB phosphatase activity [242]. Tie2 activation is a potential therapeutic avenue in diabetic macular edema. Unfortunately, in clinical trials, AKB-9778 treatment failed to achieve the primary endpoint [243], however, it was well-tolerated in patients and reached secondary endpoints. Further application of this inhibitor has been proposed in delaying tumour growth and preventing metastasis by stabilising tumour vessels [244].

PTPs remain challenging targets so it will be essential to understand specific regulatory mechanisms, signalling functions and protein–protein interactions to develop new approaches, such as allosteric modulators. Additionally, knowledge of substrates may reveal additional modalities to manipulate PTP activity, or further targets that mediate adhesive processes in disease. Finally, the key roles for RPTPs in cell adhesion control and EMT raises the possibility of targeting their extracellular domains, for example, with monoclonal antibodies [245].

## Concluding remarks

The recent explosion of proteomics datasets has enabled us to visualise the extensive connections between PTPs, core adhesive complexes and signalling pathways. Their involvement ranges from core scaffolding components within adhesive complexes to dynamic regulators of tyrosine phosphorylation. Although challenging to study, in part due to a lack of specific tools and a need to understand upstream phosphorylation, the PTPs are clearly central to the coordinated regulation of cell adhesion. Individual PTPs associate with multiple complexes, suggesting they could mediate cross-talk between adhesion types. Moreover, PTPs can confer precise, spatiotemporal control through distinct subcellular localisations and local control by upstream signals such as ROS. There are no general rules for how PTPs impact on adhesive processes. Just as PTPs can be both oncogenic and tumour suppressive, they can differentially regulate processes such as EMT. This highlights the need to understand the functions of each individual PTP in defined contexts. PTPs have historically been ‘undruggable' targets, however, the recent development of allosteric inhibitors for SHP2 highlights the importance of understanding regulatory mechanisms to inform drug development. Many open questions remain. Multiple PTPs are associated with the adherens junction cadherin–catenin module. Do PTPs carry out redundant functions or do they fine tune the phosphorylation code within specific complexes? Several PTPs interact with adhesive complex components, yet are likely to be excluded from the core complex, in particular the RPTPs. Additionally, they receive feedback from adhesions. Do PTPs play a role in delimiting adhesions or controlling their size? We anticipate that phosphoproteomics and interactome studies in 3D models such as organoids, as well as super-resolution microscopy, will uncover the interplay between PTPs and cell adhesions, as well as their roles in signalling to elicit transcriptional or metabolic changes.

## Abbreviations

BCAR3: breast cancer anti-estrogen resistance 3
CSPGs: chondroitin sulfate proteoglycan
ECM: extracellular matrix
EMT: epithelial to mesenchymal transitions
ER: endoplasmic reticulum
FAK: focal adhesion kinase
GAPS: GTPase-activating proteins
GEF: guanine exchange factor
HSPGs: heparan sulfate proteoglycans
JAMs: junctional adhesion molecules
MEFs: mouse embryonic fibroblasts
PTPs: protein tyrosine phosphatases
SFKs: Src family kinases
SH2: Src Homology 2
VE: vascular endothelial
VEGF: Vascular endothelial growth factor
ZO: zonula occludens
